# Measuring Perceptual Consciousness

**DOI:** 10.3389/fpsyg.2017.02320

**Published:** 2018-01-09

**Authors:** Marjan Persuh

**Affiliations:** Department of Social Sciences, Human Services and Criminal Justice, Borough of Manhattan Community College, City University of New York, New York, NY, United States

**Keywords:** consciousness, unconscious perception, visual perception, task performance, response bias, objective measures, subjective measures

## Introduction

Conscious perception is typically assessed with either objective or subjective measures (Seth et al., 2008). Measures are considered objective if conscious perception is estimated from performance in a discrimination task; inability to discriminate between stimuli is taken as evidence that participants had no conscious perception (Hannula et al., 2005). Measures are considered subjective if participants report their visual experience on each trial (Sergent and Dehaene, 2004; Del Cul et al., 2007). One type of subjective measures consists of metacognitive judgments; the relationship between metacognition and perceptual awareness is a matter of debate (Fleming and Lau, 2014; Jachs et al., 2015) and I will not discuss these measures further. Likewise, I will not discuss post-decision wagering approaches as they are affected by the participants' risk aversion (Schurger and Sher, 2008). Proponents of subjective measures stress that objective measures (discrimination) provide only task performance and are not suitable for capturing visual experience (Lau, 2008). The major objection against subjective measures is contamination by response bias. Because it has been argued that participants can perform discrimination in the absence of perceptual awareness, many researchers currently favor subjective measures. In this paper, I show that objective measures (discrimination) and subjective measures (detection) are similar and both measure task performance. I further propose that task performance can be used as a valid measure of conscious perception.

## Objective measures

To estimate conscious perception with objective measures, participants are typically asked to discriminate between stimuli. Perceptual consciousness is thus operationalized as the ability to discriminate between visual stimuli (Eriksen, 1960). Because biased responding can influence overall accuracy, a bias free signal detection measure, d' or sensitivity, is usually calculated. If d' is not significantly different from zero, it follows that there was no conscious perception of stimuli. This is considered by many the most rigorous procedure, yet it has been criticized on several grounds (Reingold and Merikle, 1988, 1990).

The major technical criticism is that to demonstrate the absence of conscious perception, one needs to prove the null-hypothesis which is theoretically not possible (MacMillan, 1986). In practice however, this can be achieved by setting sufficiently strict criteria for statistical power, although admittedly this is somewhat arbitrary. Alternatively, one can apply a Bayesian statistical evaluation and calculate evidence for zero conscious perception (Dienes, 2014). Several additional complicating issues have been raised. For example, any measure should be exhaustive for all relevant aspects of conscious experience as well as exclusive for conscious perception only (Reingold and Merikle, 1988, 1990), although exhaustiveness and exclusiveness requirements are not unique to objective measures.

Even if all technical issues can be adequately addressed, we face a major conceptual issue that it seems cannot be solved easily. Critics of objective measures emphasize that with objective measures (discrimination) we only ever estimate task performance (Lau, 2008) and that this estimate is not a measure of conscious experience. In fact, one could, the argument goes, estimate performance of any animal or even a photodiode and that would not require any consideration of consciousness at all. That is correct; objective measures only estimate task performance. However, I will argue that subjective measures (detection) fare no better; they only provide a different estimate of task performance and are thus vulnerable to the same criticism as objective measures. The main reason is that in detection task one performs just another type of discrimination, usually stimulus vs. background. This fact is not well appreciated and many investigators believe that objective measures should be abandoned in favor of subjective measures that estimate conscious perception more directly (Merikle et al., 2001).

## Subjective measures

The appeal of subjective measures lies in the fact that one presumably obtains introspective reports, which unlike objective measures, are more directly related to participants' conscious visual perception (Dehaene and Naccache, 2001; Merikle et al., 2001). Three types of subjective measures have been proposed: confidence ratings, post-decision wagering, and direct reports of visibility. The exact nature of the relationship between confidence ratings about perceptual decisions or metacognitive approaches and perceptual consciousness is currently a matter of debate (Zehetleitner and Rausch, 2013; Fleming and Lau, 2014; Jachs et al., 2015; Rausch et al., 2015). Similarly, the relationship between post-decision wagering (Persaud et al., 2007) and perceptual consciousness is unclear and can be affected by the participants' risk aversion (Schurger and Sher, 2008; Fleming and Dolan, 2010). Here, I discuss only subjective measures in which participants directly report their visual experience. Participants are typically asked to report their conscious visual perception of the stimulus on every trial and trials are sorted based on perception scale. Scales vary in gradation from binary “seen,” “unseen” (Lau and Passingham, 2006; Ro, 2008) and multiple discrete levels scales (Zeki and Ffytche, 1998; Overgaard et al., 2006) to scales with continuous visibility (Sergent and Dehaene, 2004). To obtain evidence for unconscious processing, only trials on which participants report no conscious perception of the stimulus are typically analyzed. This procedure is more meaningful for consciousness studies, the argument goes, because evidence for unconscious perception is obtained only from trials on which participants state directly that they did not see the stimulus.

Although this procedure has been criticized on several fronts (Goldaimond, 1958; Eriksen, 1960), the major criticism is the possibility of biased responding. Whether or not the participant responds with “yes” on particular trial depends in part on his or her willingness to say “yes.” Because subjective reports seem much more appealing for measuring conscious perception, the problem with biased responding is acknowledged but downplayed (Merikle et al., 2001).

If this is the case should we use subjective measures? In the following section I present a line of reasoning that shows the illusory nature of subjective measures.

## The illusory nature of subjective measures

Is there a value in using subjective measures because they better capture subjective experience, which is what we are interested in? Well, let us examine this issue a little closer by looking at a participant responding in a detection task. Suppose that on each trial we present either a dim red circle or no circle and participant John is instructed to perform a detection task. We run the experiment and separate trials into two sets. Are we justified to say that we have separated trials based on John's subjective experience? What exactly happened during the experiment? We need to take a step back and examine John's ability to follow instructions in this particular task. We, as experimenters, have no direct access to John's experience of red and he has no means of having us experience the red he is experiencing. These facts are generally accepted and acknowledged by the proponents of subjective measures (Dehaene and Naccache, 2001). What we do have however, is a set of correlations. As John acquired his vocabulary, he learned that people say the word “red” when looking at strawberries, blood, and lips; he established an association between his experience of redness and arbitrary motor output “red.” In the detection task, he is simply reporting that correlation. Thus, we are only measuring his performance and his reports have nothing that tells us directly about his actual experience. By the same token, a parrot can learn a correlation between its visual experience and some arbitrary motor output. We should be able to obtain the same type of “subjective” reports from animals. In other words, an organism can only report correlations and we can only measure task performance. Although consciousness researchers generally accept the private nature of a first-person experience, subjective reports, such as “I see a red circle”, are taken at face value.

Importantly, John has no direct knowledge of the outside world. He is classifying his perceptual experiences regardless of whether we call the measure objective or subjective. No matter how the question is posed, language does not give John any special powers to make his report more subjective. When only trials on which John reported no conscious perception are analyzed, we are essentially taking only misses and discarding hits. This approach creates two problems. First, classifying a trial as a miss depends on John's ability to discriminate the signal (a red circle) from the noise (monitor background) as well as on his criterion placement. Because the distribution of hits and misses depends on arbitrary criterion placement, such trials should not be interpreted in isolation (Schmidt, 2015). Second, an awareness measure is usually combined with an indirect measure (e.g., priming) in studies of unconscious perception. By selecting a subset of trials (misses) *post-hoc*, researchers may obtain above chance performance on an indirect measure for a purely statistical reason, the regression to the mean phenomenon (Shanks, 2017).

In a typical discrimination task, both hits and misses are used for calculating task performance. Although objective measures are not immune to response bias, a bias free signal detection measure of sensitivity (d') provides one way of analyzing task performance data. By treating a detection task as a discrimination task, one can calculate d' and obtain an objective measure of performance. Selecting only a subset of trials from a detection task (misses) doesn't suddenly make a measure subjective. We have only different types of tasks and associated task performances. The only question is how to properly analyze data from such tasks. One could still argue that misses in a detection task constitute a special, “No experience” category and are therefore more suitable for consciousness studies.

## Is the “no experience” category special?

To appreciate that direct visibility reports are no more subjective than objective measures, let us examine a detection task a bit closer. Because in a detection task, unlike in a discrimination task, one category is “No experience,” it seems natural to conclude that such subjective reports are closer to what the participant is experiencing, because this category presumably contains no phenomenology. But such a conclusion is unwarranted. One can run the same task but instead ask participants to rate the visibility on a popular perceptual awareness scale (PAS), which increases the number of response options from two to four. Using PAS, subjects report their visual experience using four ratings: (1) No experience, (2) Brief glimpse, (3) Almost clear experience, (4) Absolutely clear experience. Now the participants have a chance to report their experience on a finer scale. Suddenly what was in the previous task categorized as “No experience” is now distributed among additional categories, such as “Brief glimpse.” In fact, each conscious experience is unique and the participant is making fine discriminations and categorizing them into discrete classes based on the response options provided. The detection task is a discrimination task because our visual experiences (qualia) are perceptual discriminations. Otherwise we would have to conclude that using subjective visibility reports on PAS includes subjective and objective measures; comparing trials with ratings 1 and 2 would constitute a subjective measure, since it contains category “No experience” and comparing trials with ratings 2 and 3, an objective measure, since a “No experience” category is not included. Importantly, the “No experience” category does not represent a lack of phenomenology. A participant engaged in a perceptual detection task is experiencing monitor background when reporting “No experience” of the target stimulus. Instead of treating the “No experience” category as a special case, one could even argue that the categories “No experience” and “Brief glimpse” are closer in perceptual space than the categories “Brief glimpse” and “Almost clear experience.”

If we are left only with task performance, can we even study consciousness? After all, one can, for example, measure d' for a metal detector. But I think we can. The reason is that measuring d' at the personal level, in an experiment with manual or verbal non-speeded responding, taps into different information processing than measuring d' for an isolated subsystem. If we accept that visual experiences are visual discriminations at the personal or more generally, at the organismal level, then discrimination performance is a valid measure of perceptual consciousness. By analogy, living systems display a set of characteristics that together constitute “life.” There is no magic, no vital force, just chemicals organized and interacting in a specific, self-organizing manner, that give rise to the emergent property we call “life.” One could argue that measuring growth of an organism or parameters of its reproduction is not meaningful, because one can measure growth of a crystal in solution or parameters of its “reproduction,” but that is clearly not the case. At the level of an organism measuring specific processes carries a different meaning.

## Conclusions and implications for consciousness research

The debate whether objective or subjective measures provide a better estimate of conscious perception has revolved largely around the issues of response bias and inadequacy of relying on task performance alone. I suggest that subjective measures do not assess the subjective character of our visual experience any better than objective measures. It follows that the “subjective” character of subjective measures is illusory and that subjective measures, like objective measures, estimate only performance on a discrimination task. The issue of response bias, associated with any discrimination task, needs to be addressed with a proper data analysis technique and signal detection theory provides a bias free measure of performance. An awareness measure is typically combined with an indirect measure of stimulus processing (e.g., priming); this is a simple dissociation paradigm, which requires demonstration of stimulus processing in the absence of conscious perception. Which type of discrimination task is the most appropriate to measure conscious perception will vary with the particular experimental question. In some studies, awareness of stimulus location or color might be of interest; in others, investigators might assess whether participants can detect the stimulus. Or, in a different experiment, one could compare two types of discrimination performances and ask for example, whether feature awareness automatically results in location awareness. More generally, in some experiments, d' might be the most appropriate measure; in others, visibility ratings might be particularly useful. Different behavioral tasks used for measuring perceptual consciousness, including detection, are all different versions of perceptual discrimination, because our visual experiences are perceptual discriminations.

## Author contributions

The author confirms being the sole contributor of this work and approved it for publication.

### Conflict of interest statement

The author declares that the research was conducted in the absence of any commercial or financial relationships that could be construed as a potential conflict of interest.
